# A Study of Neck Circumference in Metabolic Syndrome and Correlation With Cardiometabolic Risk Factors

**DOI:** 10.7759/cureus.91655

**Published:** 2025-09-05

**Authors:** Rutva Harish Fatnani, Srikanth Narayanaswamy, Shwetha BM

**Affiliations:** 1 Department of General Medicine, Ramaiah University of Applied Sciences, Bengaluru, IND

**Keywords:** cardiometabolic risk factors, hypertension, metabolic syndrome, neck circumference, obesity

## Abstract

Background

Metabolic syndrome increases the risk of developing chronic health issues, particularly those related to the heart and glucose metabolism. Neck circumference (NC) has emerged as a simple and effective anthropometric marker reflecting upper-body adiposity, with strong associations to insulin resistance and visceral fat distribution.

Materials and methods

A total of 160 people participated in this cross-sectional study, 80 of whom had metabolic syndrome and the remaining 80 of whom were age-matched controls. Participants were recruited from the Medicine and Allied Departments at M.S. Ramaiah Hospital. Metabolic syndrome was diagnosed based on commonly accepted clinical criteria. Participants underwent clinical evaluation, anthropometric assessments, as well as laboratory tests such as fasting blood sugar (FBS) and lipid profile. NC was measured and analyzed for its correlation with various cardiometabolic risk factors.

Results

The mean NC was much greater in the case group compared to controls. NC was strongly positively correlated with triglycerides, low-density lipoprotein (LDL) cholesterol, dimensions of the waist and hips, blood pressure, FBS, total cholesterol, and body mass index (BMI) (*p* < 0.001). Gender-specific neck circumference thresholds for predicting metabolic syndrome were determined by the Youden index; these thresholds were 36.76 cm for males and 34.75 cm for females, with both showing good specificity.

Conclusion

NC is significantly connected to the elements of metabolic syndrome. As a low-cost and easy-to-measure parameter, NC shows potential as a practical metabolic syndrome screening tool, especially in resource-limited settings.

## Introduction

Metabolic syndrome and cardiovascular risk

Obesity, central, raised triglyceride levels, reduced levels of high-density lipoprotein (HDL) cholesterol, high blood pressure (BP), and impaired glucose regulation are all components of the metabolic syndrome, a group of associated metabolic diseases that can make cardiovascular disease (CVD) and type 2 diabetes more likely [1]. These components often present differently across genders [2].

Individuals with metabolic syndrome are approximately three times more likely to experience myocardial infarction, largely due to excessive adipose tissue, particularly in the visceral region [3]. This fat accumulation disrupts fatty acid metabolism, leading to higher concentrations of atherogenic lipids, such as low-density lipoprotein (LDL) cholesterol and triglycerides [4]. Insulin resistance, resulting from elevated free fatty acid levels, further impairs glucose metabolism and accelerates the onset of diabetes [5].

CVDs remain at the top of the global health agenda [6]. CVDs are responsible for approximately 17.9 million deaths annually, accounting for about 31% of global mortality, with the World Health Organization (WHO) reporting that 85% of these are due to heart attacks and strokes [7].

Because fat distribution is a crucial factor in cardiovascular risk, the body mass index (BMI), despite its widespread usage, is not a good diagnostic of obesity [8]. The visceral or fat on the upper body, especially so, is strongly linked with CVD compared to the peripheral or lower-body fat [9]. Several organizations, including the International Diabetes Federation (IDF) and the National Cholesterol Education Program (NCEP), have proposed criteria for diagnosing metabolic syndrome, but these may not be universally applicable across different ethnic populations [10]. Additionally, in environments with limited resources, access to comprehensive metabolic testing is often inadequate, underscoring the need for simple, reliable screening tools [11].

Neck circumference (NC) has proved to be a useful anthropometric variable, which depicts the upper body's fat distribution [12]. It can be measured easily and has been shown to have great correlations with insulin resistance, arterial stiffness, visceral adiposity, and cardiometabolic risk [13]. Research indicates that NC can be more indicative of metabolic syndrome than the conventional measurements, such as BMI, especially among women and in settings where high-tech diagnostic equipment is not an option [14].

Considering these findings, it can be hypothesized that there exists a connection between the enlargement of the NC and the occurrence of metabolic syndrome [15]. NC may be an effective, inexpensive, and convenient screening strategy to identify people at high cardiovascular risk, particularly in underserved groups [16,17].

Aims and objectives

This investigation aims to assess the NC as a potential indicator of metabolic syndrome and to investigate its connection to recognized cardiometabolic risk factors. 

## Materials and methods

Study design and population

The M.S. Ramaiah Hospitals served as the site of this investigation between February 2021 and October 2022. The study was conducted among the adults visiting the outpatient departments of medicine and allied branches and their admitted inpatients. The participants of the research were separated into two categories: the first category consisted of patients with a confirmed diagnosis of metabolic syndrome who fulfilled the requirements of this syndrome, and the second category consisted of age-matched persons who did not meet the criteria of metabolic syndrome, 80 patients in each category. The NCEP's Adult Treatment Panel III (NCEP-ATP III) served as the basis for the classification.

Metabolic syndrome diagnosis

The metabolic syndrome was diagnosed using the NCEP-ATP III guidelines, which were modified for the Indian population. A participant with at least three of the following five parameters was labeled to have the metabolic syndrome The criteria included waist circumference (WC) greater than 90 cm in men and greater than 80 cm in women; triglycerides levels ≥150 mg/dL; HDL cholesterol <40 mg/dL in men and <50 mg/dL in women; BP ≥ 130/85 mmHg or being on anti-hypertensive medications; and fasting blood sugar (FBS) ≥100 mg/dL or being on anti-diabetic medications. Besides these parameters, NC was also taken in all the subjects, and the measurement correlated with the other risk factors for cardiometabolic diseases using the Pearson correlation coefficient.

Inclusion and exclusion criteria

All adult participants included those who provided informed consent. The exclusion criteria were used to reduce confounding in the study; these included pregnancy, thyroid disorders, neck surgical history, malignancy, type 1 diabetes, and previous myocardial infarction.

Sample size calculation

Using a normal statistical procedure, the sample size was established by comparing the BMI means of the case and control groups. The formula used was:

Sample size = (mean BMI of cases - mean BMI of controls) × 1.96 (pooled standard error)

This ensured adequate power for detecting significant relationships between metabolic syndrome and NC.

Study methodology

Once the consent was taken and the exclusion criteria implemented, all the participants were then thoroughly clinically assessed. This commenced by collecting demographic data and a complete medical history that gave information on the existence of disorders like diabetes, elevated BP, stroke, and ischemic heart disease. Anthropometric measurements were then recorded using standardized procedures. With a person standing erect, weight was measured using an analog or digital scale, whereas height was evaluated in a stadiometer with the participant in the Frankfurt plane. NC was measured just beneath the laryngeal prominence, hip circumference at the broadest point of the buttocks, and the waist circumference was taken at the midpoint between the iliac crest and the lower rib margin. In measuring BP, a sphygmomanometer was used once the participant was at rest after five minutes, and to enhance the success of this procedure, it was recommended that the participant should not take tea, coffee, or smoke tobacco products right before the reading was taken.

Laboratory investigations

A clearance of the absence of a history of myocardial infarction was accomplished by an electrocardiogram (ECG) performed in all the participants. To carry out a comprehensive profiling of metabolic profiles, samples of blood in the veins were drawn after a minimum of eight hours of fasting during the night. The direct MgCl2 method was used to check the cholesterol levels of LDL, and HDL cholesterol was calculated by the direct measure method. The oxidase-esterase-peroxidase was used to govern the amount of total cholesterol, and the enzymatic endpoint measured triglycerides. The glucose oxidase-peroxidase method for measuring fasting blood glucose was employed. According to the outcomes of the given investigations, individuals included were split into dual categories: one with metabolic syndrome and the other without metabolic syndrome, as per NCEP-ATP III standards.

Data analysis

Data analysis was made by the programs Microsoft Excel (Microsoft® Corp., Redmond, WA) and SPSS (IBM SPSS Statistics for Windows, IBM Corp., Armonk, NY). The predictive power of NC and other variables was assessed utilizing a variety of mathematical analyses, including independent t-tests, Mann-Whitney U-tests, chi-square tests, and descriptive statistics. Pearson relationship coefficients and study of receiver operating characteristic (ROC) curves.

## Results

FBS was markedly elevated in cases (173.6 mg/dL) compared to controls (100.6 mg/dL), indicating poor glycemic control (p < 0.001). The lipid profile showed substantial differences: HDL (good cholesterol) was lower in cases (35.5 mg/dL vs. 47.9 mg/dL). LDL, total cholesterol, and triglycerides were significantly higher in cases. These differences suggest a more atherogenic lipid profile in cases. Both the systolic BP (SBP) and diastolic BP (DBP) were noticeably elevated in certain situations. Cases showed pre-hypertensive to hypertensive readings (139.13/83.85 mmHg). Controls maintained normal BP ranges (119.38/74.55 mmHg). Table 1 reveals the significant differences between cases and controls in some parameters. BMI was significantly higher in cases (27.4 ± 5.007) compared to controls (22.1 ± 2.206), indicating that cases were overweight on average, while controls maintained a normal weight (p < 0.001).

**Table 1:** Comparison of case and control means for clinical parameters BMI: body mass index, DBP: diastolic blood pressure, FBS: fasting blood sugar, HDL: high-density lipoprotein, LDL: low-density lipoprotein, SBP: systolic blood pressure

Parameter	Cases (mean ± SD)	Controls (mean ± SD)	p-value
Waist-hip ratio	0.990 ± 0.057	0.996 ± 0.547	0.559
BMI (kg/m²)	27.4 ± 5.007	22.1 ± 2.206	<0.001
FBS (mg/dL)	173.6 ± 56.94	100.6 ± 27.36	<0.001
HDL (mg/dL)	35.5 ± 11.6	47.9 ± 7.3	<0.001
LDL (mg/dL)	101.65 ± 45.45	69.62 ± 24.78	<0.001
Cholesterol (mg/dL)	161.93 ± 45.15	112.71 ± 30.83	<0.001
Triglycerides (mg/dL)	167.78 ± 66.07	99.04 ± 28.27	<0.001
SBP (mmHg)	139.13 ± 14.71	119.38 ± 9.9	<0.001
DBP (mmHg)	83.85 ± 9.29	74.55 ± 7.09	<0.001
Neck circumference (cm)	37.4 ± 3.19	33.35 ± 2.5	<0.001

NC was higher in cases (37.4 ± 3.19) compared to controls (33.35 ± 2.5). All parameters except the waist-hip ratio (WHR) revealed significant changes in terms of statistics (p < 0.001). The cases demonstrate a constellation of cardiometabolic risk factors, including elevated BMI, poor glycemic control, dyslipidemia, and higher BP. This pattern suggests a higher cardiovascular risk profile in the case group. Table 2 shows a significant positive correlation of NC with waist circumference, hip circumference, and BMI in both cases and controls (p < 0.001).

**Table 2:** Correlation coefficient between neck circumference and cardio-metabolic risk factors (anthropometric parameters) BMI: body mass index

Parameter	Cases (r)	p-value	Controls (r)	p-value
Waist circumference	0.558	<0.001	0.449	<0.001
Hip circumference	0.409	<0.001	0.430	<0.001
BMI	0.346	0.002	0.408	<0.001

For both cases and controls, the average NC for men was 38.39 ± 3.0367 and 34.031 ± 2.5979, respectively, with p < 0.001. The average circumference of the neck for females in cases and controls was 36.05 ± 2.94 and 32.32 ± 1.99, respectively (p < 0.001). Table 3 demonstrates a favorable relationship between NC and several metrics, including hip and waist circumferences and the ratio of waist to hip, LDL cholesterol levels, and BMI. This significant correlation suggests that NC could effectively predict metabolic syndrome.

**Table 3:** Pearson's correlation coefficient between cardio-metabolic risk variables and neck circumference *Indicates p < 0.05. **Indicates p < 0.001. BMI: body mass index, DBP: diastolic blood pressure, FBS: fasting blood sugar, HDL: high-density lipoprotein, LDL: low-density lipoprotein, SBP: systolic blood pressure, WHR: waist-hip ratio

Parameter	Cases (r)	p-value	Controls (r)	p-value
SBP	0.016	0.88	-0.054	0.635
DBP	-0.009	0.938	-0.032	0.776
FBS	0.112	0.322	0.281	0.012
HDL	0.038	0.736	-0.026	0.817
LDL	0.306	0.006*	0.350	<0.001**
Total cholesterol	0.161	0.154	0.108	0.339
Triglycerides	-0.025	0.825	-0.025	0.824
Waist circumference	0.558	<0.001**	0.449	<0.001**
Hip circumference	0.409	<0.001**	0.430	<0.001**
WHR	0.272	0.015*	-0.355	<0.001**
BMI	0.346	0.002*	0.408	<0.001**

**Figure 1:**
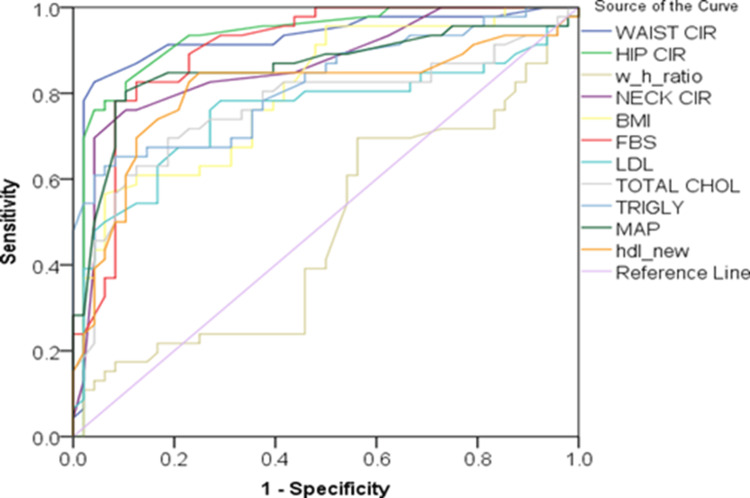
Comparison of different metrics as indicators of male metabolic syndrome Created by authors. BMI: body mass index, FBS: fasting blood sugar, HDL: high-density lipoprotein, HIP CIR: hip circumference, LDL: low-density lipoprotein, MAP: mean arterial pressure, NECK CIR: neck circumference, TOTAL CHOL: total cholesterol, TRIGLY: triglycerides, WAIST CIR: waist circumference, w_h_ratio: waist-hip ratio

Figure 1 illustrates that waist circumference, hip circumference, NC, BMI, FBS, and LDL show strong diagnostic accuracy, while triglycerides and MAP demonstrate lower predictive performance.

Table 4 suggests that waist circumference, hip circumference, FBS, NC, and MAP have the highest diagnostic accuracy (AUC > 0.85), while the WHR shows no discriminative ability (AUC = 0.477, p = 0.705).

**Table 4:** Comparing several measures to predict male metabolic syndrome BMI: body mass index, FBS: fasting blood sugar, HDL: high-density lipoprotein, HIP CIR: hip circumference, LDL: low-density lipoprotein, MAP: mean arterial pressure, NECK CIR: neck circumference, TOTAL CHOL: total cholesterol, TRIGLY: triglycerides, WAIST CIR: waist circumference

Variables in test results	AUC	Standard deviation	Asymptotic significance	Asymptotic confidence interval - lower bound	Asymptotic confidence interval - upper bound
WAIST CIR	0.922	0.032	0.000	0.860	0.985
HIP CIR	0.929	0.028	0.000	0.873	0.985
Waist-hip ratio	0.477	0.061	0.705	0.358	0.597
NECK CIR	0.863	0.039	0.000	0.786	0.940
BMI	0.806	0.044	0.000	0.720	0.893
FBS	0.900	0.033	0.000	0.835	0.964
LDL	0.753	0.054	0.000	0.648	0.858
TOTAL CHOL	0.769	0.052	0.000	0.668	0.870
TRIGLY	0.821	0.044	0.000	0.735	0.907
MAP	0.860	0.042	0.000	0.777	0.942
HDL	0.803	0.049	0.000	0.707	0.900

Additionally, the area beneath the curve was displayed by the ROC curve analysis for a number of factors, like waist circumference, hip circumference (AUC = 0.929), FBS, and NC.

The ROC curve for NC shows a region beneath the curve of 0.863, with a standard error of 0.039 and (p < 0.001).

**Figure 2:**
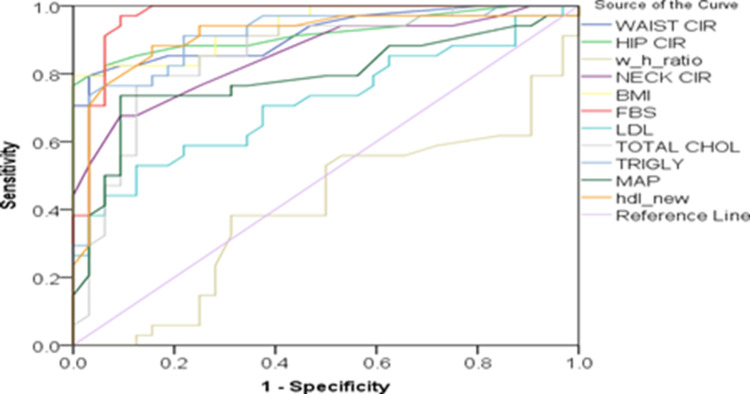
Comparison of different metrics as indicators of female metabolic syndrome Created by authors. BMI: body mass index, FBS: fasting blood sugar, HDL: high-density lipoprotein, HIP CIR: hip circumference, LDL: low-density lipoprotein, MAP: mean arterial pressure, NECK CIR: neck circumference, TOTAL CHOL: total cholesterol, TRIGLY: triglycerides, WAIST CIR: waist circumference, w_h_ratio: waist-hip ratio

Most other parameters showed good discriminative ability (AUC > 0.8), except for the WHR, which proved to be a poor diagnostic indicator (AUC = 0.477). Figure 2 depicts that hip circumference, waist circumference, FBS, NC, and MAP demonstrate high diagnostic performance with ROC curves nearing the top-left corner, while the WHR shows poor discriminative ability.

Table 5 suggests that FBS, BMI, HDL, triglycerides, and waist and hip circumference exhibit excellent diagnostic accuracy (AUC > 0.90, p < 0.001), while NC, total cholesterol, and MAP show good performance (AUC 0.78-0.86), and WHR demonstrates poor discriminative ability (AUC = 0.406, p = 0.191).

**Table 5:** Comparing several metrics to predict female metabolic syndrome BMI: body mass index, FBS: fasting blood sugar, HDL: high-density lipoprotein, HIP CIR: hip circumference, LDL: low-density lipoprotein, MAP: mean arterial pressure, NECK CIR: neck circumference, TOTAL CHOL: total cholesterol, TRIGLY: triglycerides, WAIST CIR: waist circumference

Variables in test results	AUC	Standard deviation	Asymptotic significance	Confidence interval asymptotically - lower bound	Asymptotic confidence interval - upper bound
WAIST CIR	0.918	0.034	<0.001	0.851	0.985
HIP CIR	0.920	0.036	<0.001	0.849	0.990
Waist-hip ratio	0.406	0.071	0.191	0.267	0.545
NECK CIR	0.855	0.046	<0.001	0.764	0.946
BMI	0.937	0.028	<0.001	0.882	0.991
FBS	0.968	0.022	<0.001	0.925	1.000
LDL	0.715	0.064	0.003	0.590	0.840
TOTAL CHOL	0.855	0.048	<0.001	0.761	0.950
TRIGLY	0.910	0.037	<0.001	0.838	0.983
MAP	0.788	0.059	<0.001	0.673	0.904
HDL	0.911	0.040	<0.001	0.834	0.989

NC area beneath the curve in females (0.855), with a standard error of 0.39 (p < 0.001). Most metabolic parameters demonstrate good to excellent discriminative ability with AUC > 0.8, except for LDL (AUC = 0.715), which shows fair accuracy, and MAP (AUC = 0.788), showing moderate accuracy. Notably, the WHR (AUC = 0.406) demonstrates poor diagnostic utility and is not statistically significant (p = 0.191). These findings collectively suggest that anthropometric measurements (except WHR) combined with metabolic parameters provide the most reliable diagnostic utility for identifying cases, indicating the presence of multiple cardiometabolic risk factors that would require comprehensive management strategies.

Table 6 displays the NC cut-off values that were established in the current investigation for the purpose of predicting metabolic syndrome. The NC threshold value to predict metabolic syndrome, determined by using the Youden index, showed a NC cut-off of 36.76 cm in males, with ROC analysis showing 76.1% sensitivity and 89.6% specificity. With a 90.6% specificity and a sensitivity of 67.5%, the NC cut-off was 34.75 cm in females. In addition to supporting the usefulness of NC as a metabolic syndrome screening test, these gender-specific cutoffs show high diagnostic qualities.

**Table 6:** Male and female neck circumference cut-off values

Gender	Cut-off value (cm)	Sensitivity (%)	Specificity (%)
Males	36.76	76.1	89.6
Females	34.75	67.5	90.6

## Discussion

The results presented in the study bring into focus the close relationship between NC and several cardiometabolic risk factors, making it an accurate anthropometric measurement for identifying metabolic syndrome. In all metrics of metabolism, the case group had higher values compared to the control group, with significant differences observed in all measurements. ROC curve analysis revealed strong diagnostic accuracy for several variables, including FBS, BMI, hip circumference, and NC, with AUC values exceeding 0.8 in both genders. These results align with earlier studies demonstrating an association between NC and cardiovascular risk [18,19].

Gender-specific NC cut-off values were also reported; a cut-off of 36.76 cm in males showed a specificity of 89.6%, while 34.75 cm in females showed a specificity of 90.6%. These thresholds are consistent with values reported in other studies, with minor variations that may be attributed to ethnic and population-specific differences [20,21]. This highlights the importance of establishing region- and ethnicity-specific NC reference standards.

Furthermore, NC demonstrated significant positive correlations with waist circumference, BMI, and lipid variables, supporting its potential as a screening tool for metabolic risk. Notably, NC showed a moderate but statistically significant correlation with waist circumference in the case group (r = 0.558, p < 0.001), indicating that while the relationship is not very strong, it is clinically meaningful and consistent with previous studies of anthropometric indices. This suggests that NC could reasonably supplement, or in certain settings even substitute, traditional measures of central adiposity. These observations are in line with research emphasizing the metabolic importance of upper-body subcutaneous fat [22]. Interestingly, WHR demonstrated poor diagnostic performance, with an AUC of 0.406 and an insignificant p-value (0.191), contradicting earlier models and supporting more recent evidence questioning the reliability of WHR as a sole diagnostic tool [23].

The metabolic syndrome profile, characterized by elevated FBS, dyslipidemia, and hypertension, was present in the case group and is especially concerning in South Asian populations, who are known to develop metabolic complications at lower BMI thresholds [24]. Insulin resistance and systemic inflammation are likely key mechanisms linking NC to various aspects of metabolic syndrome [25]. This association is further supported by additional population-based studies that endorse the utility of NC as a screening measure across diverse demographic settings [26].

Given its simplicity, cost-effectiveness, and high specificity, NC measurement is especially valuable in low-resource clinical environments. Based on these findings, incorporating NC into routine screening protocols is recommended to facilitate early diagnosis and management of metabolic syndrome, as suggested by multiple studies.

Limitations of the study

The main shortcomings are the comparatively limited sample size. No inferences can be drawn because of the cross-sectional form of the study on the causes of NC and cardiometabolic risk variables.

## Conclusions

Several variables combine to form metabolic syndrome, which relates to each other and increases the risk of acquiring heart disease considerably. Additionally, it carries severe clinical consequences, significant economic costs, and a detrimental effect on living quality, and requires timely prevention. NC in the study was revealed to positively correlate with some of the important anthropometric indices that included the BMI and waist circumference, implying the possibility of using it as a proxy degree of obesity. Additionally, NC was strongly connected with increased levels of triglycerides, LDL cholesterol, total cholesterol, and fasting blood glucose, as well as high BP, confirming its relationship with cardiometabolic risk factors. NC proves to be a viable screening measure due to its ease of use, affordability, and the simplicity with which it can be performed in resource-limited settings with limited access to full-scale testing. These results justify NC measurements in regular clinical training to assist in the early detection, risk stratification, and therapy for those at increased risk for heart disease.
